# Bacterial and Host Determinants of Group B Streptococcal Vaginal Colonization and Ascending Infection in Pregnancy

**DOI:** 10.3389/fcimb.2021.720789

**Published:** 2021-09-03

**Authors:** Alyssa Brokaw, Anna Furuta, Matthew Dacanay, Lakshmi Rajagopal, Kristina M. Adams Waldorf

**Affiliations:** ^1^Center for Global Infectious Disease Research, Seattle Children’s Research Institute, Seattle, WA, United States; ^2^Department of Global Health, University of Washington, Seattle, WA, United States; ^3^Department of Obstetrics & Gynecology, University of Washington, Seattle, WA, United States; ^4^Department of Pediatrics, University of Washington, Seattle, WA, United States; ^5^Department of Obstetrics and Gynecology, University of Washington and Sahlgrenska Academy, University of Gothenburg, Gothenburg, Sweden

**Keywords:** group B streptococcus, bacteria, pregnancy, colonization, vagina, placenta, fetus, preterm birth

## Abstract

Group B streptococcus (GBS) is a gram-positive bacteria that asymptomatically colonizes the vaginal tract. However, during pregnancy maternal GBS colonization greatly predisposes the mother and baby to a wide range of adverse outcomes, including preterm birth (PTB), stillbirth, and neonatal infection. Although many mechanisms involved in GBS pathogenesis are partially elucidated, there is currently no approved GBS vaccine. The development of a safe and effective vaccine that can be administered during or prior to pregnancy remains a principal objective in the field, because current antibiotic-based therapeutic strategies do not eliminate all cases of invasive GBS infections. Herein, we review our understanding of GBS disease pathogenesis at the maternal-fetal interface with a focus on the bacterial virulence factors and host defenses that modulate the outcome of infection. We follow GBS along its path from an asymptomatic colonizer of the vagina to an invasive pathogen at the maternal-fetal interface, noting factors critical for vaginal colonization, ascending infection, and vertical transmission to the fetus. Finally, at each stage of infection we emphasize important host-pathogen interactions, which, if targeted therapeutically, may help to reduce the global burden of GBS.

## Introduction

Group B Streptococcus (GBS) (also called *Streptococcus agalactiae*) is a gram-positive, β-hemolytic, and chain-forming bacterium that can asymptomatically colonize the human vaginal and gastrointestinal tracts. However, during pregnancy, GBS can become highly invasive and pathogenic to the fetus and mother, leading to adverse outcomes. Invasive GBS infections during pregnancy result in preterm birth (PTB), stillbirth, and fetal death. The fetus and neonate are highly susceptible to GBS, causing sepsis, pneumonia, and meningitis which, in severe cases, leads to long-lasting sequelae that can affect the child’s central nervous system and lung function (Bianchi-Jassir et al., 2017; Madrid et al., 2017; Seale et al., 2017b). In the mother, GBS infections can lead to maternal sepsis and postpartum endometritis (Hall et al., 2017). The complex pathogenesis of maternal and infant GBS infection arises due to the large arsenal of the bacterium’s virulence factors, which can vary widely across strains and undergo altered expression depending on the host niche. Some virulence factors are implicated in a single disease outcome, such as the role of HvgA in neonatal meningitis (Tazi et al., 2010). Other virulence factors are more broadly associated with GBS pathogenesis, including the hemolysin (also called β-hemolysin/cytolysin) (Pritzlaff et al., 2001; Liu et al., 2004; Hensler et al., 2005; Lembo et al., 2010); the molecular basis of which is the GBS pigment, or granadaene and has direct cytotoxic effects against many types of host cells (Whidbey et al., 2013; Gendrin et al., 2015; Whidbey et al., 2015; Boldenow et al., 2016; Armistead et al., 2020a). Host-pathogen interactions that alter the balance between vaginal tract colonization and GBS invasive disease ascending into the uterus are beginning to be elucidated (Vornhagen et al., 2018a). Further investigation of the GBS bacterial factors enabling colonization of the vagina and invasive disease will facilitate the design of novel GBS prophylactics and treatments that directly target pathogenesis. In this review, we summarize the seminal studies and recent advances that define our understanding of GBS disease pathogenesis at the maternal-fetal interface, focusing on the interplay between bacterial virulence factors and host defenses that ultimately leads to GBS clearance or invasion.

## Methods

### Search Strategy and Selection Criteria

To identify recent and seminal GBS research relating to maternal colonization, ascending infection, and fetal injury, literature searches were performed using Google Scholar and PubMed Central. Variants of the terms “pregnancy,” “preterm labor,” “premature,” “vaginal colonization,” “colonization,” “uterus,” “ascending infection,” “placenta,” “fetus,” “vaccine” were used in combination with “Group B Streptococcus” or “Streptococcus agalactiae.” The literature search was limited to articles published between 2014-2021. However, we used snowball searches of article reference list to identify studies that were seminal for recent work in the field, including work published prior to 2014.

## GBS Epidemiology and Prophylactic Measures

### Global Epidemiology of GBS Disease Outcomes

GBS colonizes the rectovaginal space in approximately 20-25% of women worldwide, with rates varying widely in different parts of the world (Seale et al., 2017a). In a meta-analysis that pooled estimates of GBS colonization from 85 countries, the prevalence of colonization varied between 7-14% in Central America and Asia to 35% in the Caribbean. Europe, North America, and Australia had similar prevalence rates of 15-20%. Interestingly, estimates in Africa varied widely with West and South Africa at 14 and 25%, respectively (Russell et al., 2017). Due to the asymptomatic nature of rectovaginal colonization, it is difficult to determine if the prevalence of GBS colonization is changing over time; however, there is evidence that invasive GBS infection of nonpregnant adults is increasing. Between 2008 to 2018 within the Active Bacterial Core surveillance network, the incidence of invasive GBS infection in nonpregnant individuals increased from 8.1 to 10.8 cases per 100,000 adults, representing 11.5% of the US population (Francois Watkins et al., 2019). These studies highlight that GBS contributes significantly to the global burden of disease, and in addition, emphasize that GBS rectovaginal colonization is common worldwide.

**Figure 1:**
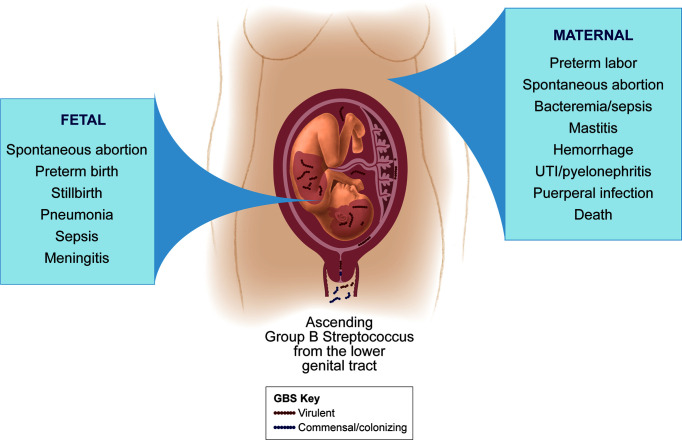
GBS disease manifestations for mothers and fetuses. GBS is considered a commensal colonizer of the maternal vaginal tract, but ascension of GBS into the uterus can lead to adverse perinatal outcomes. GBS can invade the amniotic cavity and fetus to cause a variety of disease manifestations in the fetus (left panel), some of which can occur even with a limited infection of the placental chorioamniotic membranes. Maternal GBS invasive disease is more infrequent and can vary in severity (right panel); more severe outcomes tend to occur in the setting of chorioamniotic membrane rupture and/or with maternal co-morbidities that compromise the immune response.

Although GBS vaginal colonization is asymptomatic in most women, it is of particular concern during pregnancy due to the significant risk for adverse fetal and neonatal outcomes (**Figure 1**). Maternal GBS infection is a leading cause of infection-induced PTB and stillbirth (Seale et al., 2017a). Further, in 2015 more than 20 million neonates were exposed to maternal GBS out of 140 million live births worldwide, and half of neonates born to pregnant GBS-colonized women are estimated to themselves become colonized (Seale et al., 2017a). In a conservative analysis of international data from a single year, at least 90,000 infant deaths (<3 months of age), 10,000 cases of disability in children, 57,000 fetal infections or stillbirths, and 3.5 million cases of PTB were attributed to GBS. A disproportionate burden of these GBS cases occurred in Africa, which accounts for 54% of annual GBS cases and 64% of all fetal and infant deaths (Seale et al., 2017a). In addition to its fetal and neonatal disease burden, GBS can cause serious maternal disease. Recently, it was estimated that there are at least 33,000 annual cases of maternal invasive GBS disease worldwide (Seale et al., 2017a). Another systematic literature review found that 0.38 women per 1,000 pregnancies experience invasive GBS disease with symptoms ranging from endocarditis or pneumonia to maternal death in severe cases (Hall et al., 2017). GBS invasive disease in pregnancy poses significant risks to both the fetus and mother.

Importantly, the prevalence of maternal, fetal, and neonatal disease are likely underestimated. In low-income countries that have the highest rates of invasive GBS cases, the microbiological cause of sepsis, PTB, stillbirth, or death may not be recognized if cultures are not obtained or if formalized pre- or antenatal visits are inaccessible. In addition, policies for GBS screening are highly variable both geographically and across high, middle, and low income countries. Policies regarding screening generally fall into two categories: microbiological or risk-factor based screening. Microbiological screening, sometimes known as universal screening, tests all mothers at a specific gestational age. In contrast, risk-factor based screening is more cautious in that mothers are tested only if they present with symptoms of GBS invasive disease (such as preterm labor) or if they have a history of adverse pregnancy outcomes. Screening methods, however, are not necessarily consistent among nations with similar economic status; for example the United States performs universal microbiological screening by rectovaginal swab at 35-37 weeks gestation, while Sweden and the United Kingdom utilize risk-based screening (Le Doare et al., 2017). Mothers who test positive for GBS are generally administered intravenous antibiotics during labor, called intrapartum antibiotic prophylaxis (IAP). Unfortunately IAP policies also vary due to cost or rising concerns of antibiotic resistance. Discrepancies in both screening and antibiotic use can have drastic effects on GBS case counts and birth outcomes. When symptoms present only after delivery, cases may not be attributed to GBS until late in the clinical course. Subclinical cases that can still lead to lifelong complications may remain undetected and untreated, and may never be linked to GBS infection. Additionally, by the time symptoms present in the clinic it may be too late to intervene. Rapid and low-cost testing strategies that can be implemented in low-resource settings would likely allow for more accurate estimates of the GBS burden of disease, and might provide opportunities to reduce this burden through IAP intervention.

### GBS Serotypes and Capsule Polysaccharide-Based Vaccines

Many GBS virulence factors influence a strain’s ability to colonize or cause severe disease. One key virulence factor is the sialylated GBS capsular polysaccharide (CPS), first identified by Dr. Rebecca Lancefield in the 1930s (Lancefield, 1934). To date, there are ten known capsular serotypes (Ia, Ib, II-IX), although six account for 98% of GBS colonization worldwide (Ia, Ib, II-V) (Russell et al., 2017). These same serotypes cover over 99% of overall cases, including EOD and LOD (Nanduri et al., 2019). Not unlike colonization rates and disease burden, GBS serotype frequencies vary geographically. For example, 25% of colonizing strains are serotype III worldwide, but this serotype only accounts for up to 10% of cases in Asian and African countries. Other serotypes such as V-IX are more prevalent in Africa and Southern, South-Eastern, and Eastern Asia (Russell et al., 2017). In a meta-analysis, serotype Ia and III were linked to 31 and 27% of GBS maternal invasive cases, respectively (Hall et al., 2017), while fetal and neonatal disease was dominated by serotype III which was responsible for 62% of cases. Serotypes Ia, Ib, and V are also commonly associated with EOD and LOD, but their contribution is largely dwarfed by serotype III (Madrid et al., 2017).

Although the GBS capsule is involved in a variety of immune evasion mechanisms (Marques et al., 1992; Takahashi et al., 1999; Weiman et al., 2010; Uchiyama et al., 2019), the GBS CPS conjugated to an immunogenic protein carrier is a well-studied vaccine strategy. Vaccination of pregnant rats with CPS III conjugate reduced vaginal colonization, chorioamnionitis, and vertical transmission (Chiarot et al., 2018), spurring the examination of these vaccines in human clinical trials. In nonpregnant women, this vaccine induced heightened antibody responses and delayed vaginal and rectal acquisition of GBS (Hillier et al., 2019). A major limitation of CPS-based vaccines is the need to confer protection against multiple serotypes, and thus multivalent CPS vaccine strategies are of high interest. A Novartis/GSK trivalent vaccine (Novartis/GSK GBS3) comprised of CPS Ia/Ib/III conjugate is considered safe in pregnant women, elicited high maternal antibody titers and the antibodies were placentally transferred to infants (Swamy et al., 2020). Unfortunately, a trivalent vaccine still does not include all clinically-relevant serotypes and could lead to selection of non-vaccine strains through capsule switching, a phenomenon that has been observed in many high-risk populations and among ST-17 strains (Bellais et al., 2012; Meehan et al., 2014; Neemuchwala et al., 2016; Nagano et al., 2019). Further, a recent study identified patients that were co-infected by multiple GBS serotypes at once (Jisuvei et al., 2020), which may enable capsule switching. Thus, a hexavalent CPS conjugate vaccine derived from serotypes Ia, Ib, II, III, IV, and V would cover more clinically-relevant strains (Bianchi-Jassir et al., 2020). A hexavalent CPS conjugate vaccine (Pfizer GBS6) induced the production of opsonophagocytic antibodies in vaccinated rhesus macques and protected murine infant pups from lethal GBS challenge upon maternal vaccination (Buurman et al., 2019). This Pfizer GBS6 vaccine was also well-tolerated in healthy adults and induced high antibody titers (Absalon et al., 2021), but its immunogenicity and efficacy has not been examined in clinical trials in pregnant women. Compared to whole CPS-based vaccines that are difficult to produce, an alternative strategy might utilize a more specific CPS epitope that can be chemically synthesized. One group has utilized X-ray crystallography and epitope mapping to design a synthetic CPS III glycan-conjugate vaccine (Michon et al., 2006; Carboni et al., 2017; Carboni et al., 2020), which appears to confer similar levels of protection to whole CPS conjugate (Oldrini et al., 2020). However, to address the challenge of capsule switching, highly technical and intensive X-ray crystallography studies would be needed to identify relevant epitopes for the other CPS types. A more recent development that might simplify this problem is the production of GBS strains that express chimeric CPS. In a mouse model, maternal vaccination with a chimeric CPS V/IX conjugate resulted in the protection of pups from infection by GBS serotypes V or IX (Campisi et al., 2021). Owing to the limited immunogenicity of CPS, the difficulty of attaining serotype coverage, and challenges related to CPS manufacturing, other virulence factors may serve as better or alternate vaccine targets.

### GBS Sequence Types and Virulence Factor Epidemiology

Aside from its serotype distinction, GBS can also be clustered genetically through a technique known as multi-locus sequence typing. This is performed by sequencing seven housekeeping genes for any GBS strain-of-interest, and the combination of these seven genomic loci generates a sequence type (ST) (sometimes called clonal complex or CC) (Jones et al., 2003). Among a global and ecologically diverse sample of strains, four major sequence types were found, ST-1, ST-17, ST19, and ST23 (Jones et al., 2003). Of these, ST-1 and ST-19 were largely associated with asymptomatic carriage, ST-23 was common among both asymptomatic carriers and invasive disease cases, and ST-17 serotype III strains were attributed to neonatal invasive infections (Jones et al., 2003). In contrast, a study of 225 invasive isolates from Taiwan implicated both ST-1 and ST-17 as invasive isolates causing maternal and neonatal disease (Tsai et al., 2019). Importantly, ST-17 strains are emerging internationally and are associated with an increased propensity for neonatal meningitis and antibiotic resistance (Tazi et al., 2010; Teatero et al., 2016; Kao et al., 2019). However, antibiotic resistance is also emerging among non-ST-17 strains (Hays et al., 2016). This increase in antibiotic resistance poses a significant threat, as IAP is currently the only FDA-approved GBS treatment for pregnant women and the only preventative measure against vertical transmission of GBS. Hypervirulent strains, including ST-17, have also recently been isolated from tilapia (Evans et al., 2009; Chen, 2019), and there are rising concerns that tilapia-adapted GBS might serve as a zoonotic route for human transmission (Sun et al., 2016). These challenges emphasize important gaps in screening for antibiotic resistant isolates, which could inform intrapartum antibiotic prophylaxis. In addition, hypervirulent ST-17 GBS may have emerged in humans following zoonotic transmission from cows (Bisharat et al., 2004), and thus improved GBS surveillance among animal reservoirs might provide insight into the emergence of clinically significant isolates and consequent challenges.

## GBS Vaginal Colonization and Persistence

### Maternal GBS Colonization Is a Risk Factor for Subsequent Disease

During infection of the maternal-fetal interface, GBS first encounters the maternal rectovaginal tract. In fact, one of the best-defined risk factors for fetal or neonatal adverse outcomes is maternal colonization (Bianchi-Jassir et al., 2017). Although only about 25% of women are colonized (Russell et al., 2017), GBS is often considered a member of the host microbiota because carriage is usually asymptomatic. However, temporal carriage of vaginal GBS can be incredibly variable; wherein one patient may be persistently colonized whereas another may experience recurring or intermittent colonization (Brzychczy-Wloch et al., 2014; Sroka-Oleksiak et al., 2020). Additional studies are needed to understand how colonization varies temporally and across populations, particularly among pregnant and nonpregnant women. Current findings suggest that vaginal colonization is regulated by many overlapping bacterial and host factors. Gastrointestinal and rectal GBS colonization are likely to modulate maternal colonization by directly seeding GBS into the vaginal tract (Meyn et al., 2009). For this review, we will focus on vaginal colonization as this location is the primary source of GBS that can lead to maternal-fetal disease. Unless otherwise specified, GBS colonization or carriage refers to that within the vaginal tract.

### Interactions Between the Vaginal Microbiome and GBS Are Complex

The vaginal microbiota is composed of a wide array of taxa that varies geographically, by racial or ethnic group, throughout the menstrual cycle, and during pregnancy (Sroka-Oleksiak et al., 2020). Many taxa, including *Prevotella bivia*, *Veillonella* spp., *Eubacterium siraeum*, and *Staphylococcus* spp. have been associated with GBS vaginal colonization in nonpregnant women. In contrast, bacterial vaginosis (BV)-associated bacterium 1 and 2, *Prevotella* genogroups 3 and 4, *Dialister* species type 2, and *Megasphaera* species type 1 are associated with a lack of GBS (Rosen et al., 2017). Many of these latter taxa are associated with bacterial vaginosis (BV), a heterogeneous vaginal microbial dysbiosis (Onderdonk et al., 2016). However, a recent small study of nonpregnant Chinese women observed increased *Prevotella* and *Megasphaera* spp. abundance among women who were GBS-colonized (Mu et al., 2019). Other studies using rats and mice have uncovered relationships between GBS colonization and the presence of BV-associated bacteria such as *P. bivia* (Cools et al., 2016) and *Gardnerella vaginalis* (Gilbert et al., 2021). Clearly, the relationships between GBS and other vaginal taxa are complicated. Conflicting findings across studies may be due in part to variation in vaginal microbiome composition, which is common across human cohorts and when comparing laboratory animals in different facilities; discrepancies in culturing and sequencing methods further complicates the comparison of such studies. Although majority of findings regarding GBS’ interaction with other microbes are associative, GBS co-colonization with *Candida albicans* observed clinically (Cools et al., 2016) is supported mechanistically by observations of interaction between the GBS BspA adhesin and the *C. albicans* hyphae-specific surface protein Als3 (Rego et al., 2016). Whether the presence of any of these taxa alter GBS colonization rates in humans is unknown, as well as the mechanisms by which colonization may be altered. Future studies should interrogate changes in niche remodeling, nutrient availability and the immune environment that may occur alongside shifts in microbiome composition. It is possible that a combination of these factors contributes to the variable and fluctuating rates of GBS carriage.

### Vaginal Lactobacilli: Key Species That Limit GBS Colonization and Persistence

The healthy vaginal tract maintains an acidic pH due to the *Lactobacillus* species that metabolize glycogen and produce lactic acid (Boskey et al., 2001). In reproductive-age women, the microbiome can be categorized into 5 community state types (CSTs I-V) based on the dominating *Lactobacillus* species. Mechanisms by which Lactobacilli restrain GBS colonization at the microbiome-level remain unclear, but multiple studies have partially defined the interactions between different *Lactobacillus* species and GBS. Lactobacilli can have antagonistic effects on the growth of GBS and host cell interactions *in vitro* (Bodaszewska et al., 2010; Ortiz et al., 2014; Marziali et al., 2019; He et al., 2020). While in human observational vaginal microbiome studies, the relationship between *Lactobacillus*-replete microbiomes and GBS colonization is less clear (Kubota et al., 2002; Altoparlak et al., 2004; Daniel Johannes Rönnqvist et al., 2006; Brzychczy-Wloch et al., 2014; Rick et al., 2017), there is evidence that reduced taxonomic diversity and *Lactobacillus* dominance are possible mechanisms of microbiome-based protection against GBS during pregnancy (Sroka-Oleksiak et al., 2020). An exception is CST IV, which encompasses a more diverse and dysbiotic assortment of non-*Lactobacillus* anaerobes accompanied by increased vaginal pH (Ravel et al., 2011). This CST can be further differentiated into IV-A and IV-B (Gajer et al., 2012), and CST IV-A has been linked to increased GBS risk and appears to be *Streptococcus* and *Prevotella*-dominant (Rosen et al., 2017; Brotman et al., 2018). These data suggest that Lactobacilli alone do not explain differences in GBS carriage status, although this genus likely affects GBS colonization in concert with other vaginal taxa. A better understanding of what promotes *Lactobacillus*-replete vaginal microbiomes during pregnancy is needed, and how these changes alter the vaginal environment including immune responses remains to be determined. Interestingly, a subset of specific pathogen-free mice appears to possess *Lactobacillus*-dominant vaginal flora that might more closely mimic that of the healthy human vagina. Preliminary evidence suggests that this murine CST may impair GBS persistence (Vrbanac et al., 2018). A mechanistic understanding of the environmental or genetic cues that culminate in the development of this human-like murine vaginal microbiome could allow for more relevant studies of GBS vaginal colonization and persistence in mice.

### GBS Adaptation in the Vaginal Tract Favor Virulence

GBS is frequently described as an opportunist or pathobiont due to its ability to transition from an asymptomatic colonizer to a potent pathogen (Armistead et al., 2019). During this transition, GBS must adapt to its new niche in order to successfully colonize and persist in the vaginal tract. Luckily, GBS encodes a plethora of two-component systems (TCS), each comprised of a sensor histidine kinase and DNA-binding response regulator (denoted by S and R, respectively), that grant the ability to respond to diverse environmental stimuli.

One well-studied GBS TCS, CovR/S (also known as CsrR/S), responds to low pH (Park et al., 2012), which is likely encountered in the healthy human vaginal tract. CovR/S is a master regulator of GBS virulence, and more than a hundred genes undergo transcriptional changes in response to CovR. Interestingly, one phenotype of GBS lacking this TCS (denoted as Δ*covRS* or Δ*covR*) is hyper-adhesion, which occurs due to increased expression of cell surface adhesins (Lamy et al., 2004; Park et al., 2012). CovR/S also regulates expression of the *cyl* operon (Jiang et al., 2005), which encodes factors required for the biosynthesis of GBS’ characteristic hemolysin (Whidbey et al., 2013; Whidbey et al., 2015; Armistead et al., 2019; Armistead et al., 2020a). This virulence factor is a pro-inflammatory cytotoxin that is essential for many facets of GBS disease including pneumonia, sepsis, meningitis, PTB, and invasive infection of adults (Nizet et al., 1996; Nizet et al., 1997; Gibson et al., 1999; Doran et al., 2002; Doran et al., 2003; Hensler et al., 2005; Whidbey et al., 2013; Whidbey et al., 2015; Boldenow et al., 2016; Armistead et al., 2020a; Armistead et al., 2020b), and the pro-inflammatory nature of this factor is likely the reason hyperhemolytic GBS strains have reduced vaginal persistence (Gendrin et al., 2015; Patras et al., 2015). Another more recently characterized TCS that is important for GBS vaginal carriage is SaeR/S, which was identified by a transcriptomic screen using mice that were vaginally colonized with GBS. The SaeR/S system regulates the transcription of surface adhesin PbsP during murine vaginal colonization, and in fact *pbsP* is upregulated more than 250-fold during murine vaginal colonization and GBS lacking PbsP exhibit reduced vaginal persistence (Cook et al., 2018). While the extracellular signal recognized by SaeR/S is unknown, it is hypothesized to be a small peptide, potentially an antimicrobial peptide (AMP) present in the vaginal tract. Future studies should aim to establish the relevance of SaeR/S in human vaginal colonization and persistence, and define the roles of other SaeR-regulated factors.

In contrast to CovR/S and SaeR/S, other TCSs confer protection against host defenses encountered in the vaginal tract, prolonging GBS survival. DltR/S is involved in cell wall lipoteichoic acid (LTA) maintenance, and importantly, loss of this system alters LTA expression and increases GBS susceptibility to AMPs common in the reproductive tract (Poyart et al., 2001; Poyart et al., 2003). LiaR/S also allows evasion of antimicrobial defenses by regulating cell wall synthesis and cell membrane modification, permitting GBS to evade AMPs (Madanchi et al., 2020). Although these TCS may contribute to vaginal persistence, their roles have yet to be interrogated *in vivo*.

### GBS Factors That Facilitate Vaginal Adhesion and Persistence

GBS encodes numerous surface adhesins and invasins that interact with host epithelia and promote infection, although the data for some of these surface proteins is more convincing than for others. BspA, a member of the antigen I/II protein family, mediates binding to vaginal epithelial cells through interactions between its own V domain and the host gp340 surface receptor (Rego et al., 2016). Further, GBS surface β protein has recently been shown to interact with epithelial CEACAM1 and 5 receptors (van Sorge et al., 2021), which are common in the vagina and uterus, respectively (Islam et al., 2018). As previously discussed, PbsP promotes GBS vaginal persistence in mice (Cook et al., 2018). Apart from direct binding to host epithelial cell receptors, GBS can also interact with host extracellular matrix (ECM) components. The serine-rich repeat glycoprotein Srr1 binds to keratin 4 or fibrinogen (Samen et al., 2007; Mistou et al., 2009; Sheen et al., 2011; Seo et al., 2013); in murine models, this interaction contributes to cervicovaginal persistence in a fibrinogen- and Srr1-latch domain-dependent manner (Wang et al., 2014). FbsC (also called BsaB) similarly binds the host ECM *via* laminin and fibrinogen to allow cervicovaginal adhesion of GBS (Park et al., 2012; Buscetta et al., 2014). Other surface proteins, such as pili, are also important during GBS colonization. For example, bacterial competition studies show that pilus-null GBS are outcompeted by an isogenic wild-type strain during vaginal colonization of mice (Sheen et al., 2011). Although some GBS surface proteins have not been studied extensively *in vivo*, the above factors appear have been directly linked to the vaginal tract.

### The Vaginal Immune Response Can Restrict GBS

**Figure 2:**
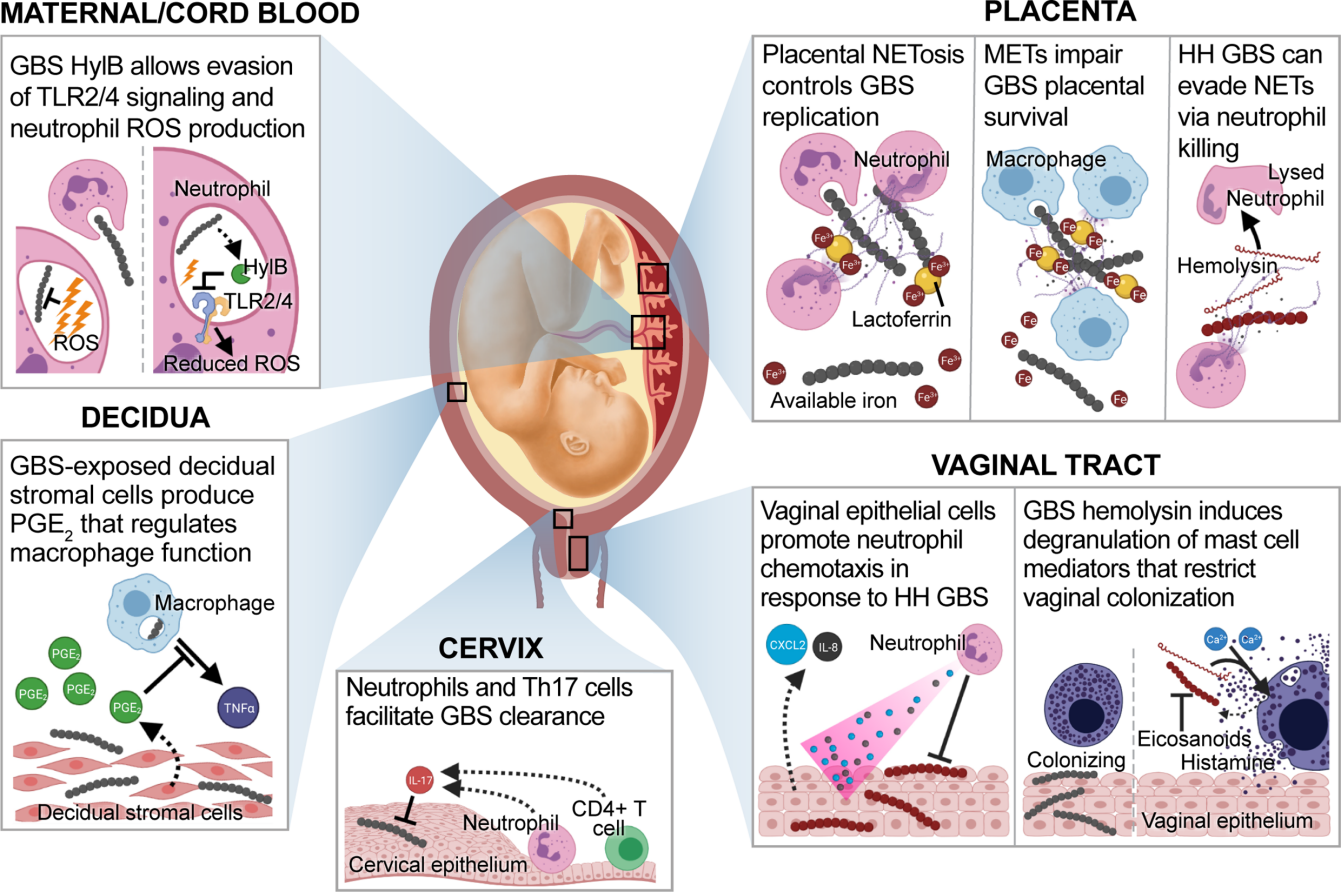
GBS-directed host immune responses at the maternal-fetal interface. During GBS infection at the maternal-fetal interface, a variety of immune cell interactions can occur. In the vaginal tract, epithelial cells exposed to hyperhemolytic (HH) GBS secrete C-X-C motif chemokine ligand 2 (CXCL2) and interleukin (IL)-8 which mediate GBS elimination through the recruitment of neutrophils (Patras et al., 2013). Whereas wild-type GBS colonize the lower genital tract in the presence of mast cells, HH GBS induce mast cell degranulation in a hemolysin and calcium-dependent manner. This results in the release of pre-formed mast cell mediators, such as eicosanoids and histamine that mediate GBS clearance (Gendrin et al., 2015). During ascending infection, cervical Th17 cells that produce IL-17 are associated with GBS clearance (Patras et al., 2015). Decidual stromal cells exposed to GBS produce prostaglandin E_2_ (PGE_2_), which suppresses macrophage tumor necrosis factor alpha (TNF-α) production (Rogers et al., 2018). Placental neutrophils produce neutrophil extracellular traps (NETs) that immobilize GBS and facilitate clearance *via* phagocytosis and nutritional immunity. NET-contained lactoferrin sequesters ferric iron (Fe^3+^), resulting in GBS growth arrest (Kothary et al., 2017). HH GBS evade placental NETs by hemolysin-mediated killing of neutrophils (Boldenow et al., 2016). Macrophages control placental GBS replication through a similar mechanism, and macrophage extracellular traps (METs) contain similar products (Doster et al., 2018). Finally, neutrophils in maternal and cord blood phagocytose GBS and produce reactive oxygen species (ROS) to mediate bacterial killing. However, GBS expressing hyaluronidase (HylB) inhibit this mechanism by interfering with toll-like receptor 2 and 4 signaling. Downstream effects of this immune dampening suppress ROS production and facilitate GBS survival (Coleman et al., 2021).

GBS must also overcome the vaginal immune response in order to achieve persistent colonization. Vaginal tract colonization induces the recruitment of numerous immune cells, which can facilitate bacterial clearance through diverse mechanisms (**Figure 2**, vaginal tract). The production of IL-1β, IL-6, IL-8, IL-23, IL-17, IFN-γ, and TNF-α are associated with reduced GBS colonization (Patras et al., 2013; Carey et al., 2014; Patras et al., 2015; Sweeney et al., 2020). These proinflammatory mediators are primarily produced by neutrophils recruited through IL-8, CXCL1, and CXCL2 (Patras et al., 2013; Carey et al., 2014; Patras et al., 2015), macrophages (Carey et al., 2014), and vaginal epithelial cells (Patras et al., 2013). In addition, vagina-resident mast cells contribute to GBS clearance through the hemolysin-induced release of pre-formed inflammatory mediators, including histamine (Gendrin et al., 2015). However, the roles of natural killer and dendritic cells (DCs) in vaginal immune responses are incompletely understood. In mice, vaginal clearance is associated with Th1, Th2, and Th17 responses (Carey et al., 2014; Patras et al., 2015). Studies using B cell-deficient or neonatal Fc receptor-null mice suggest mucosal B cells are involved in the clearance of vaginal GBS (Baker et al., 2017). Further, increases in serotype-specific anti-CSP antibody titers and opsonophagocytic activity (OPA) correlate with reduced colonization rates (Kwatra et al., 2015). Importantly, multiple studies have identified strain-level differences in immune responses that may partially explain differences in strain and serotype colonization of the vagina (Patras et al., 2015; Sweeney et al., 2020).

### Novel Prophylactics to Prevent Vaginal Colonization

The ability to prevent maternal vaginal colonization would eliminate a significant proportion of GBS morbidity and mortality. GBS interactions in the vaginal tract highlight important bottlenecks and are defined by specific host-pathogen interactions that could be targeted by therapeutics. Although CPS conjugate vaccination is correlated with reductions in GBS carriage, serotype coverage and capsule switching are still major concerns. Thus, the development of other novel strategies remains a priority.

The vaginal microbiome is malleable, and therefore therapies capable of inducing changes in composition might successfully restrict or eliminate GBS. Although it remains unclear whether Lactobacilli inhibit GBS colonization *in vivo*, probiotic *Lactobacillus* species have potential as a novel therapeutic. In mice, serial vaginal inoculation with probiotic *L. reuteri* CRL1324 confers partial protection against GBS, and this effect is mediated at least in part by mucosal immunity (De Gregorio et al., 2015; De Gregorio et al., 2016). Human clinical trials for oral probiotic Lactobacilli are also encouraging. A small randomized controlled trial found that administration of *L. rhamnosus* GR-1 and *L. reuteri* RC-14 during the third trimester reduced GBS rectovaginal colonization rates at delivery (NCT01577108) (Ho et al., 2016). In another study, the same probiotics reduced the abundance of vaginal GBS and BV-associated taxa (Liu et al., 2020). In addition, the probiotic *L. salivarius* CECT9145 also reduced GBS rectovaginal colonization during pregnancy (Martín et al., 2019). Though these studies sound promising, others have observed no protective effect (Farr – NCT03008421, Sharpe – SCRD42020216531) (Olsen et al., 2018; Farr et al., 2020; Sharpe et al., 2021), and thus larger studies are required to confirm efficacy. It is also worth noting that due to differences in vaginal CST and microbiome composition, probiotics may not serve as a universal prophylactic against GBS. Furthermore, it is unknown how stable or long-lasting probiotic-induced changes to the microbiome are. As such, it is essential that follow-up studies are performed across international cohorts and in combination with substantial characterization of the vaginal microbiome to better inform the effects of oral probiotic use.

GBS utilizes many surface adhesins and invasins during the vaginal colonization stage, which have been targeted by vaccines (**Table 1**). Maternal vaccination with a chimeric protein derived from six variants of the pilus type 2a backbone subunit (6xD3) resulted in protection of neonates against lethal challenge with GBS that carry type 2a pili (Nuccitelli et al., 2011); however, vaginal colonization was not interrogated. A similar mouse model determined that maternal vaccination with a C5a peptidase-containing vaccine protected neonates against lethal challenge and, furthermore, prevented subsequent vaginal colonization of the dam (Santillan et al., 2008; Santillan et al., 2011). BibA also holds promise as a vaccine, as mucosal vaccination with recombinant BibA resulted in protection of mice against both systemic and vaginal challenge (dos Santos et al., 2020). Moreover, this vaccine also conferred protection to neonatal mice in a maternal vaccination model (Santi et al., 2009). These studies highlight that adhesin-based vaccines are encouraging, although these strategies are more nascent than CPS conjugate vaccines and thus many remain in preclinical stages. Even so, these vaccines are not without limitations. A major challenge in targeting adhesins is their functional redundancy, and thus future studies need to test which adhesins are dispensable *in vivo* and which are not. In addition, while the surface adhesins are better conserved than CPS, GBS adhesins may vary by strain or across ST lineages. Consequently, adhesin-based strategies may still need to target multiple GBS factors in order to prevent colonization by all relevant strains. Despite this limitation, novel vaccines utilizing GBS adhesins and invasins antigens warrant future investigation in clinical trials, and should be considered as an attractive alternative to CPS-based vaccines.

**Table 1:** Novel in-pipeline vaccines that target GBS determinants of vaginal colonization and invasive disease.

GBS Bacterial Factor	Role in Pathogenesis	Vaccine	Study Design*	Reference
Capsule	Immune evasion at varied stages of infection	Novartis/GSK GBS3	Human (phase II: NCT02046148)	(Swamy et al., 2020)
		Pfizer GBS6	Mouse (maternal vaccination); Rhesus macaque (no challenge); Human (phase I/II: NCT03170609)	(Buurman et al., 2019; Absalon et al., 2021)
		Synthetic CPS III glycan conjugate	Mouse (no challenge)	(Carboni et al., 2020; Oldrini et al., 2020)
		Chimeric CPS V/IX conjugate	Mouse (maternal vaccination)	(Campisi et al., 2021)
Pilus	Adhesion to vaginal epithelium	6x3D	Mouse (maternal vaccination)	(Nuccitelli et al., 2011)
C5a peptidase	Complement evasion in blood; Attachment to fibronectin		Mouse (maternal vaccination)	(Santillan et al., 2008; Santillan et al., 2011)
BibA	Complement evasion in blood; Adhesion to cervical epithelium	Recombinant BibA with varied adjuvants	Mouse (maternal vaccination); Mouse (parenteral and mucosal vaccination with vaginal or lethal challenge)	(Santi et al., 2009; dos Santos et al., 2020)
SIP	Immunogenic protein	Recombinant SIP with varied adjuvants	Mouse (parenteral and mucosal maternal vaccination)	(Martin et al., 2002; Soto et al., 2019)
		SIP-expressing *Lactococcus lactis*	Mouse (mucosal vaccination)	(Diaz-Dinamarca et al., 2020)
Alpha-like proteins	Cervical cell invasion	GBS-NN/NN2	Mouse (maternal vaccination); Human (phase I nonpregnant: NCT02459262; phase I pregnant: NCT04596878)	(Stålhammar-Carlemalm et al., 2007; Fischer et al., 2021)

*All vaccinations utilized parenteral routes, unless specified as mucosal.

Finally, vaccine strategies that target GBS factors important for vaginal colonization should consider the route of administration as mucosal immunizations may better direct immune response to relevant tissue sites. Intranasal immunization of ethanol-killed whole GBS generated both robust systemic and mucosal responses and enhanced GBS clearance from the vaginal tract. In contrast, intramuscular immunization promoted systemic and mucosal antibodies response, but there was no difference in GBS vaginal colonization between vaccinated and control mice (Baker et al., 2017). Additionally, studies on the exceptionally well-conserved surface immunogenic protein (SIP) highlight important lessons for GBS vaccination. First, parenteral or mucosal vaccination of dams with recombinant SIP led to neonatal protection from GBS invasive disease (Martin et al., 2002). Second, a subcutaneous vaccination of SIP with AbISCO-100 adjuvant resulted in both humoral and cellular responses that protected against GBS vaginal colonization, whereas the vaccine adjuvanted with aluminum hydroxide resulted in only a weak humoral response. Serum or T cell transfer from vaccinated mice significantly reduced vaginal GBS burden in naïve mice, with T cells showing slightly higher efficacy than serum (Soto et al., 2019). This study emphasizes the importance of novel adjuvants in the formulation of a successful vaccine. Finally, mucosal vaccination may be more effective in stimulating immune cells in the vaginal tract; however, mucosal delivery is challenging. A third study by this group demonstrated a novel use for *Lactococcus lactis* as a delivery system for recombinant SIP, which again resulted in a strong humoral and cellular response that aided in vaginal clearance (Diaz-Dinamarca et al., 2020). GBS vaccines targeting SIP with an optimized adjuvant represent a promising platform to reduce GBS vaginal colonization.

## GBS Ascending Infection

### Ascension Of GBS From the Vaginal Tract May Require Transient Interaction With the Cervix

Most pathogens that cause intra-amniotic infections are vaginal tract commensals, highlighting the importance of ascending infection as a mechanism of invasive disease during pregnancy (Vornhagen et al., 2016; Vornhagen et al., 2018a; Romero et al., 2019). As a nonmotile bacterium, it is puzzling that GBS is able to ascend from the colonized vagina to the uterus. However, multiple mechanisms have been implicated in this stage of infection. GBS can promote the loss of vaginal epithelial barrier function by stimulating vaginal exfoliation (Vornhagen et al., 2018a). Additionally, GBS can interact with epithelial cells of the cervix using its diverse adhesins and invasins. Thus, cervical cell adhesion and invasion may promote GBS ascending infection into the uterus. Some adhesins, such as FbsC and Srr1, exhibit redundant function in the vagina and cervix (Buscetta et al., 2014; Jiang and Wessels, 2014; Wang et al., 2014). In contrast, BibA mediates complement evasion in the blood but also appears to mediate cervical cell adhesion (Santi et al., 2007), although this was not explicitly demonstrated in their murine model of ascending infection. GBS Alps also permit interaction with the cervical epithelium, as their binding to host α_1_β_1_ integrins or glycosaminoglycans allows cervical cell invasion *in vitro* (Bolduc et al., 2002; Baron et al., 2004; Bolduc and Madoff, 2007). The ability to invade cervical cells would confer protection from the extracellular host immune response. Although little is known regarding cervical immunity during GBS infection, IL-17-producing neutrophils or CD4^+^ T helper cells (Th17) appear to mediate GBS clearance within the murine cervix (Patras et al., 2015) (**Figure 2**, cervix). The roles of other immune cells at this site, including CD8^+^ T cells that may mediate clearance of intracellular GBS, remain undefined. As the murine cervix is small, *in vivo* studies of cervical immunity and infection are challenging and may be better suited for human studies or large animal models.

### The Cervical Mucus Plug: A Barrier to Ascension

Whether direct interaction with the cervical epithelium is required for ascending infection or not, GBS must pass through the cervix to gain entry to the uterus. During pregnancy, a cervical mucus plug (CMP) is formed in the cervix to separate the lower genital tract from the uterus. The CMP is unique to human pregnancy and is made of viscoelastic material that serves as a physical barrier. However, varied permeability, size, and mucosal adhesion is thought to correlate with risk of uterine infections that may induce PTB (Becher et al., 2009; Smith-Dupont et al., 2017; Lacroix et al., 2020). Such changes may be induced by the dysbiotic vaginal microbiota through the secretion of bacterial vaginosis-associated mucinases (Olmsted et al., 2003; Hoang et al., 2020). In addition to serving as a physical barrier, CMPs contain a variety of antimicrobial compounds including cytokines, lactoferrin, lysozyme, and AMPs (Hein et al., 2002; Lee et al., 2011; Arko et al., 2012). Thus, the CMP may be intrinsically antimicrobial and serve as a checkpoint in preventing vaginal pathogens from ascending through the cervix to invade the uterus (Hein et al., 2001; Becher et al., 2009). One study interrogating mechanisms of protection using CMPs isolated from pregnant Danish women quantified AMPs and immune mediators present therein and found that native concentrations of AMPs (including cathelicidin, elafin, and lysozyme) in the CMP were insufficient to kill GBS *in vitro* (Vornhagen et al., 2018b). Despite this inadequacy, cytokines and chemokines from the CMPs could activate immune cells in the blood, thereby enabling the CMP to enhance complement-mediated pathogen killing as observed. Interestingly, CMPs collected from women who received antibiotics exhibited bactericidal effects that were lost upon treatment with penicillinases (Vornhagen et al., 2018b), suggesting that another protective mechanism of CMPs may be through antibiotic retention. While *ex vivo* studies of CMPs collected from women receiving antibiotics may not accurately reflect the degree of their antimicrobial nature, a combination of antimicrobial products and host factors contained in the CMPs may work in concert to synergistically inhibit bacterial ascension *in vivo*.

### GBS Hijacks Host Epithelial-Mesenchymal Transition to Facilitate Ascension of Bacteria Into the Uterus

**Figure 3:**
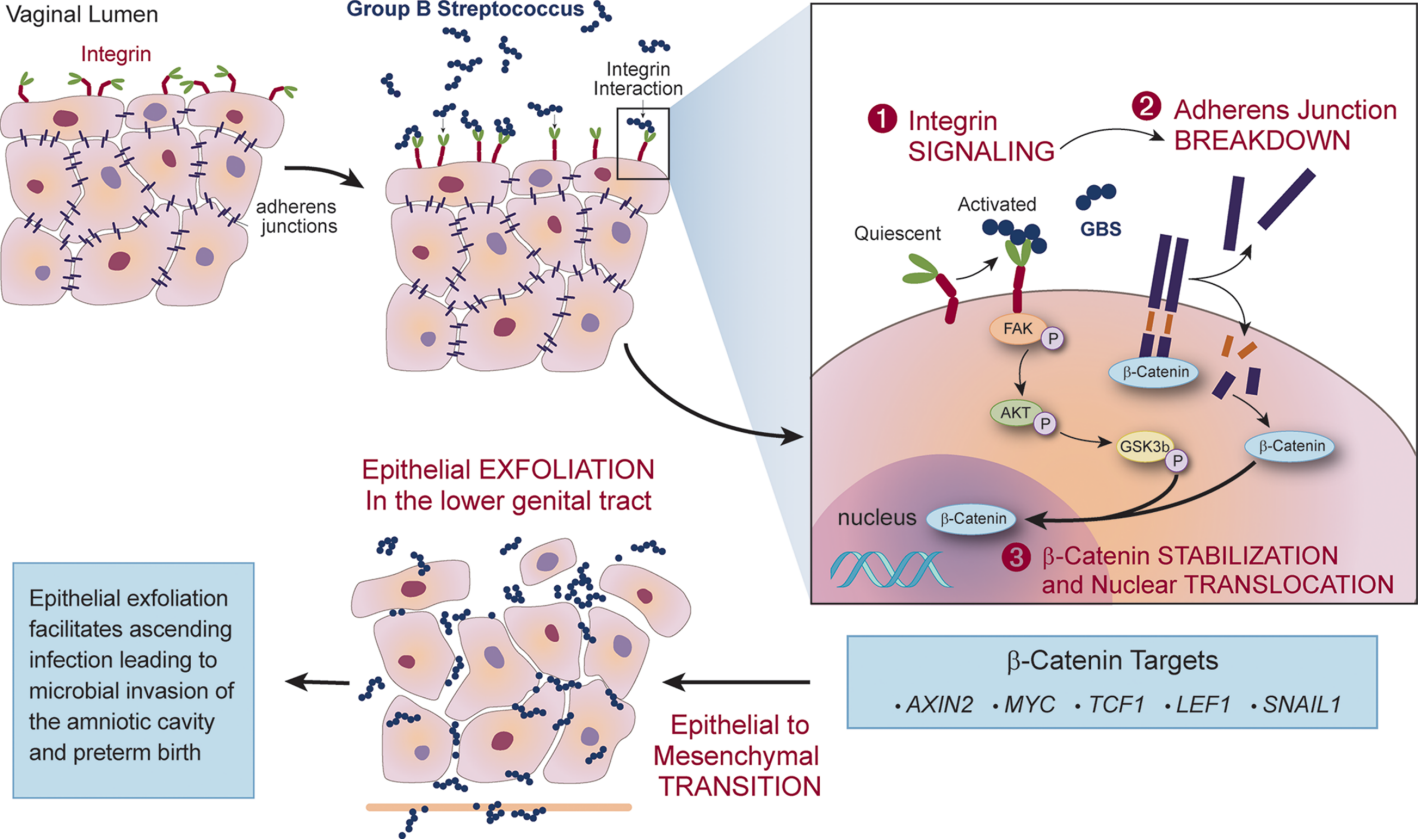
GBS exploits epithelial-mesenchymal transition (EMT) and vaginal epithelial exfoliation to permit ascending infection. During vaginal infection, GBS binds integrins on the epithelial surface and activates integrin signaling that results in the breakdown of adherens junctions. Displaced β-catenin translocates to the nucleus, activating transcriptional changes of β-catenin targets associated with EMT. Expression of these transcripts *in vivo* leads to vaginal epithelial exfoliation, which facilitates GBS ascension to the uterus and is associated with increased risk for microbial invasion of the amniotic cavity and preterm birth.

One host mechanism that can protect against an ascending infection into the uterus is vaginal epithelial exfoliation. This process is facilitated by epithelial-mesenchymal transition (EMT), a highly coordinated loss of epithelial cell tight junctions that leads to the detachment of apical cells, resulting in exfoliation of the epithelium. This phenomenon typically results in efflux of cells harboring pathogenic vaginal bacteria (Schleimer et al., 2007); however, GBS appears to exploit this response to facilitate ascending infection. During vaginal epithelial cell infection, GBS binds and activates α_1_β_1_ integrins, leading to a signaling cascade that displaces β-catenin upon the breakdown of adherens junctions. Once displaced, β-catenin can translocate to the nucleus and activate transcription of multiple genes associated with EMT, including *SNAIL1*, *MYC*, and *AXIN2* (Vornhagen et al., 2018a). *In vivo*, the expression of these transcripts results in vaginal exfoliation, which typically eradicates other vaginal pathogens. Interestingly, GBS colonization of the murine vaginal tract was unaltered despite EMT-induced vaginal exfoliation, and instead this phenomenon was associated with increased ascending infection (**Figure 3**). Administration of recombinant α_1_β_1_ integrin to GBS-infected pregnant mice rescued this phenotype, and nearly eliminated subsequent *in utero* transmission to pups and placentas (Vornhagen et al., 2018a). This strategy appears, thus far, to be unique to GBS and many aspects of this mechanism remain undefined, such as the GBS factor that activates host α_1_β_1_ integrin to promote EMT. In addition, it is unclear how EMT impacts vaginal colonization of GBS in humans.

### GBS Hyaluronidase Impedes Immune Detection and Aids in Ascension to the Uterus

Hyaluronan (HA) (also called hyaluronic acid) levels increase in the cervix throughout pregnancy, reaching their height during parturition. This glycosaminoglycan plays a structural role in the ECM, and plays additional roles in cell migration, adhesion, and inflammation through its digestion into fragments that interact with a diverse array of host receptors, including toll-like receptor (TLR) 2 and 4 (Dusio et al., 2011; Dicker et al., 2014; Mahendroo, 2019). HA is crucial for maintaining epithelial polarization and barrier function, and loss of HA is associated with ascending infection and subsequent infection-induced PTB (Akgul et al., 2014). Additionally, low molecular weight HA serves as a damage-associated molecular pattern (DAMP) that initiates TLR2 and 4-mediated inflammation during tissue injury (Termeer et al., 2002; Taylor et al., 2004; Scheibner et al., 2006). GBS exploits this axis through the secretion of hyaluronidase (HylB), which cleaves the pro-inflammatory HA DAMPs into disaccharides, which bind to TLR2/4 and block recognition of GBS ligands. This ultimately results in a dampened immune response (Kolar et al., 2015). Interestingly, GBS clinical strains isolated from cases of PTB or invasive neonatal infection had high HylB activity, and HylB activity was associated with ascending infection, reduced uterine inflammation, and increased rates of PTB in mice (Vornhagen et al., 2016). Although these studies partially define interactions in this immune evasion mechanism, the precise timing, immune mediators, and cell types involved remain unidentified.

### Novel Prophylactics During Ascending Infection

One prophylactic strategy that might prevent ascending infection is through the inhibition of Alps-mediated cervical cell invasion. This invasion phenotype is mediated by Alps N-termini, which are partially conserved across this protein family. Alps harbor tandem repeats that are immunodominant during natural infection, but the N-terminus is highly immunogenic in the absence of these repeats (Bolduc et al., 2002; Bolduc and Madoff, 2007). A peptide derived from the fusion of αC (also called AlpC) and Rib N-termini (hereby called GBS-NN) confers mice with protection against systemic lethal challenge with αC or Rib-expressing strains (**Table 1**). Antibodies against GBS-NN also inhibit epithelial cell invasion (Stålhammar-Carlemalm et al., 2007). A vaccine composed of alum-adjuvanted GBS-NN in combination with GBS-NN2, a second fusion peptide derived from N-terminal domains from the other Alps, has been designed (Minervax ApS, Denmark). This vaccine is expected to protect against nearly all GBS clinical isolates since 99% of strains express at least one Alp (Gabrielsen et al., 2017). The results from a phase I randomized double-blind, placebo-controlled study of healthy, non-pregnant women are promising (NCT02459262). Nearly all participants vaccinated with GBS-NN experienced elevated titers, especially when administered as two doses adjuvanted with alum (Fischer et al., 2021). A follow-up trial similarly tested the dual vaccine, GBS-NN/NN2, in healthy non-pregnant women (NCT03807245). This vaccine will also undergo safety and immunogenicity trials in HIV-positive and -negative pregnant women, including up to a six-month follow-up period for the women and their babies following delivery (NCT04596878). This vaccine represents a novel strategy through which ascension and cervical persistence may be targeted to ultimately prevent GBS invasive disease.

Inhibiting the trafficking of GBS from the vagina into the uterus would represent a major accomplishment in preventing fetal infection and the ensuing early-onset neonatal GBS invasive disease. However, in order to inhibit ascension, many large knowledge gaps must be filled. For example, the host factors facilitating GBS trafficking into the uterus are unknown, but may include the host vaginal microbiota, nutritional status, genetics and cervical anatomy. Further, it is exceedingly difficult to identify when GBS enters the uterus in patients, particularly because GBS invasion begins asymptomatically and symptoms only manifest at the time of a fulminant infection. Although murine models for ascending infection exist, their gestational length and fetal development are vastly different compared to humans and, thus, such models are limited in their ability to identify temporal relationships with relevance to human GBS infection. A nonhuman primate with many close similarities to human pregnancy including gestation, fetal development and sensitivity to pathogens is a better suited model of studying ascending infection and for evaluation of therapeutic strategies.

## GBS Vertical Transmission

### Role of the Immune Cells at the Maternal-Fetal Interface in Preventing Vertical Transmission

**Figure 4:**
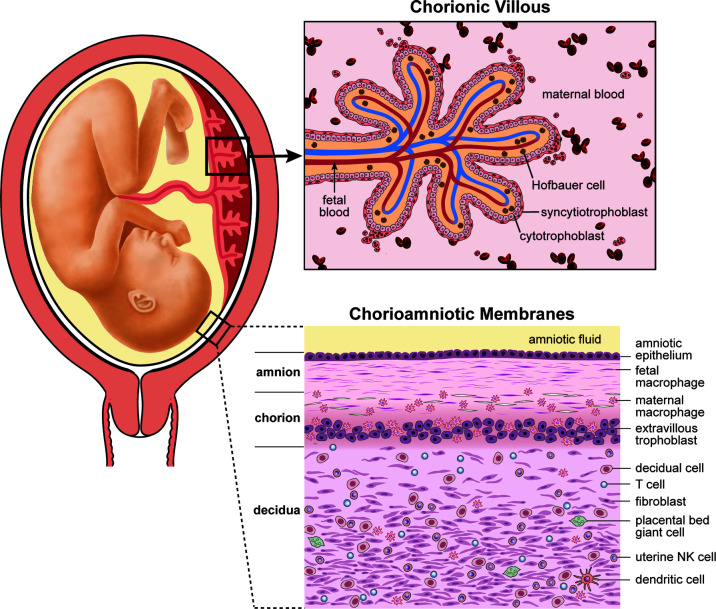
Human placental anatomy. This illustration depicts the components of the human placenta (chorionic villous tree), placental chorioamniotic membranes and the associated maternal decidua. GBS must traffic across either the decidua and chorioamniotic membranes or the chorionic villi to infect the fetus. GBS chorioamnionitis and culture-positive amniotic fluid is common in cases of fetal or neonatal invasive disease; thus, it is thought that GBS invasive disease occurs most commonly due to trafficking across the chorioamniotic membranes.

The placenta and maternal decidua serves as a multi-layered barrier rich in immune cells that separates maternal and fetal tissues (**Figure 4**). The placental disc is composed of a chorionic villous tree that is immersed in maternal blood, where gas and nutrient exchange takes place. Hematogenous pathogens can invade the placenta and gain access to the fetal circulation through the placental chorionic villi. Alternatively, maternal vaginal pathogens can ascend into the uterus through the cervix where they may either encounter the maternal decidua and chorioamniotic membranes or the placenta disc, depending on placental location (which can vary). It is unusual for the placenta to cover the cervix, a condition called placenta previa, so we will consider the most typical scenario where a vaginal pathogen would encounter the maternal decidua and subsequently, the chorioamniotic membranes on its path into the amniotic fluid. Thus, the immune cell rich decidua and chorioamniotic membranes comprise a unique immune environment that can contribute to pathogen clearance or facilitate invasion depending on pathogen virulence and host response.

A variety of immune cells are present in the maternal-fetal interface, and many aspects of their function and protective mechanisms have been studied in the context of GBS infection and chorioamnionitis (**Figure 2**, placenta/decidua). To study immune responses in the decidua, an *in vitro* approach is to use decidualized immortalized endometrial stromal cells, which mimic this cell type albeit with limitations due to immortalization. Studies using these immortalized decidual cells have determined that GBS stimulation of the mitogen-activated protein kinase (MAPK) pathway leads to NF-κB activation and programmed necroptosis through c-Jun N-terminal kinase (JNK) and p38, respectively (Flaherty et al., 2019). Using the same decidualized cell line in a co-culture study with the macrophage-like cell line THP-1, another study observed that in response to GBS, decidual stromal cells produce prostaglandin E_2_ (PGE_2_) that alters the THP-1-mediated immune response through paracrine signaling. Specifically, PGE_2_ enhanced inflammation, but inhibited THP-1 production of TNF-α and reduced phagocytosis of GBS. Conditioned medium from *ex vivo* cultured human placental membranes induced a similar effect on GBS-stimulated THP-1 cells. Mice with GBS chorioamnionitis exhibited macrophage infiltration and increased PGE_2_ (Rogers et al., 2018). Further support for paracrine signaling as a mechanism of placental immune regulation, IL-1 derived from the choriodecidua is needed for amniotic release of human beta defensin-1 during extraplacental membrane infection by GBS (Boldenow et al., 2015). Thus, host-directed therapies may be effective through direct targeting of cells that contribute to GBS infection, as well as through paracrine signaling that affects bystander cells that modulate immune responses.

Primary placental cells are perhaps an excellent model to study GBS host-pathogen interactions in the placenta. A study using primary human fetal macrophages isolated from the chorionic villous tree observed that protein kinase D signaling was critical for the GBS-induced production of inflammatory cytokines, NLRP3 inflammasome activation, and activation of NF-κB (Sutton et al., 2019). This pathway represents another host response that might be targeted therapeutically, although it is important to note that these downstream phenotypes likely contribute to both GBS clearance and immunopathology. A limitation of using primary placental immune cells is that they are unlikely to model conditions that are relevant *in vivo*. For example, when grown in isolation primary macrophages do not receive the immunologic cross-talk from stromal cells to exert paracrine effects. Thus, *ex vivo* analyses of placental immunity should seek to incorporate other cell types and signals encountered *in vivo*. One interesting approach, albeit engineered, is the novel “fetal membrane on a chip,” a system that can be easily modified to incorporate stimuli such as GBS, cytokines, hormones, prostaglandins, nutrients, or inhibitors; effluent samples can be collected for downstream analyses including Luminex or mass spectrometry (Aronoff, 2020). Overall, primary placental cells serve as an interesting model of host-pathogen interactions, but benefit from the addition of paracrine effects and novel approaches to model the anatomical complexity of the placenta.

Although *in vitro* technologies are improving, the best way to study immune responses is in a host. Thus, animal models of pregnancy-associated GBS infection and GBS chorioamnionitis are essential to inform placental immunity. In a murine model of GBS vaginal inoculation during pregnancy, neutrophil infiltrates were observed in the choriodecidua and placenta; further, these neutrophils produced neutrophil extracellular traps (NETs) that immobilized GBS and repressed GBS growth *via* lactoferrin-mediated iron sequestration (Kothary et al., 2017). Another group noted NET production in non-pregnant mice inoculated vaginally with GBS, which was dependent on GBS hemolysin (Carey et al., 2014), suggesting that GBS virulence factors may mediate this neutrophil response. However, hyperhemolytic GBS can evade NETs *via* direct neutrophil killing (Boldenow et al., 2016). Interestingly, production of extracellular traps is not a mechanism unique to neutrophils; primary placental macrophages release similar macrophage extracellular traps (METs) during GBS infection *ex vivo*. METs were produced in an ROS-dependent manner, contained a variety of matrix metalloproteases and were observed in the fetal chorioamniotic membranes (Doster et al., 2018). The production of these extracellular traps may adversely affect pregnancy outcome, as the production of matrix metalloproteases and neutrophil elastase can stimulate uterine contractions and are associated with chorioamnionitis and PTB (Sundrani et al., 2012; Tambor et al., 2013; Walsh et al., 2018). Animal models of GBS infection in pregnancy are key for elucidating complex host-pathogen responses that begin with vaginal inoculation and result in invasion of the amniotic cavity and fetus.

### GBS Trafficking Into the Amniotic Cavity

After crossing the maternal decidua and chorioamniotic membranes, GBS must traffic into the amniotic fluid and adapt to a new niche in order to subsequently infect the fetus. When GBS was grown in amniotic fluid, transcriptional changes altering metabolism of amino acids and carbohydrates was observed, which is likely related to the nutritional requirements of GBS in amniotic fluid. Further, growth in amniotic fluid also resulted in differentially expression in virulence-associated genes. Adhesins were downregulated, whereas, capsule, hemolysin, and IL-8 proteinase were upregulated, suggesting that GBS modulates its virulence factors in response to amniotic fluid (Sitkiewicz et al., 2009). Expression of the *fru2* metabolic operon which encodes factors involved in fructose metabolism (Patron et al., 2017) was induced, suggesting that this carbon source might be important for GBS survival in this niche. Although these studies highlight adaptations that GBS might undergo following microbial invasion of the amniotic cavity, the relevance of these findings *in vivo* are unclear. Recently, a transposon screen identified a novel GBS transcription factor, MrvR, that was important for survival in amniotic fluid. MrvR; deletion of *mrvR* reduced GBS survival in amniotic fluid and impaired biofilm formation. Pregnant mice vaginally-infected by MrvR-null GBS exhibited normal vaginal persistence and consistent ascending infection, but these mice did not experience PTB (Dammann et al., 2021), suggesting that MrvR may mainly promote bacterial survival *in vivo*.

Once GBS has invaded the amniotic cavity, immune recognition and inflammatory mediators contribute to the development of adverse pregnancy outcomes. In a nonhuman primate model, inoculation of GBS into the amniotic fluid increased amniotic fluid IL-1β, TNF-α, IL-6, and IL-8 cytokine/chemokine levels (Gravett et al., 1994). When these individual cytokines were inoculated into the amniotic fluid of a rhesus macaque model to interrogate the effects of each individual cytokine, only the nonhuman primates inoculated with IL-1β or TNF-α developed preterm labor (Sadowsky et al., 2006). These observations suggest that certain inflammatory cytokines are sufficient to induce preterm labor and PTB. Prostaglandins, which increase cervical softening, are also observed to increase in the amniotic fluid during GBS invasion of the chorioamniotic membranes and amniotic cavity (Boldenow et al., 2016; Coleman et al., 2021). A recent study found that higher amniotic fluid IL-6 and IL-8 concentrations are also useful predictors of fetal lung injury in a nonhuman primate model of GBS chorioamnionitis (McCartney et al., 2021). Thus, amniotic fluid cytokines and chemokines play a role in the development of adverse maternal and fetal outcomes during GBS infection.

Finally, recent studies have begun to evaluate the roles of immune cells in the amniotic fluid on bacterial clearance. Human amniotic fluid neutrophils actively phagocytose GBS *ex vivo* in a manner similar to what has been observed by neutrophils in the blood (Gomez-Lopez et al., 2017). This represents one mechanism that may contribute to reduction of GBS burden in the amniotic cavity, although this has not been established *in vivo*. Other phagocytes such as macrophages may also mediate amniotic fluid clearance, although this has not been examined. Further, immune cell subsets that contribute to pro-inflammatory cytokine responses associated with adverse outcomes remain undefined.

### GBS Virulence Factors Modulate Crucial Balance Between Chorioamnionitis and Microbial Invasion of the Amniotic Cavity

GBS can modulate host immune responses at the maternal-fetal interface to promote chorioamnionitis and microbial invasion of the amniotic cavity. An important virulence factor GBS employs to gain access to the amniotic cavity through invasion of the chorioamniotic membranes is hemolysin. In fact, GBS clinical strains isolated from the chorioamniotic membranes or amniotic fluid of women in preterm labor from a small case series exhibited a hyperhemolytic phenotype. GBS hemolysin induces the release of proinflammatory cytokines, such as IL-6, IL-8, IL-1β, and TNF-α in chorioamniotic membranes, culminating in barrier disruption (Whidbey et al., 2013). The cytotoxic activity of hemolysin is driven through intercalation of the toxin within the plasma membrane, leading to bilayer disruption followed by rapid efflux of potassium ions. In human macrophages, hemolysin-mediated potassium ion efflux triggers NLRP3 inflammasome formation and activates caspase 1 signaling, ultimately causing cell death by pyroptosis (Whidbey et al., 2015). Although caspase 1 activation can promote GBS clearance (Costa et al., 2012), the induction of robust inflammatory responses is likely detrimental during pregnancy, as the host must balance inflammation during infection to protect the mother and immunoregulatory responses to prevent preterm labor (Whidbey et al., 2015). Notably, in a pregnant murine model, hyperhemolytic GBS promoted preterm labor and significantly increased the rate of fetal death compared to an isogenic nonhemolytic strain. Intriguingly, NLRP3 inflammasome activation exacerbated GBS-mediated fetal injury. However, other host factors may also contribute to these pregnancy complications, as fetal death was still observed in NLRP3-deficient mice, albeit at lower levels than wildtype mice (Whidbey et al., 2015). In pregnant nonhuman primates that were choriodecidually infected with hyperhemolytic COH1Δ*covR*, GBS trafficked across the chorioamniotic membranes to invade the amniotic fluid and fetus more frequently than with a non-hemolytic GBS strain (COH1Δ*covR*Δ*cylE*) (Boldenow et al., 2016). Microbial invasion of the amniotic cavity was associated with robust neutrophilic infiltration of the chorioamniotic membranes and production of neutrophil extracellular traps and proinflammatory cytokines. The hyperhemolytic GBS strain subverted all of these host innate immune responses to infect the amniotic fluid and ultimately lead to adverse outcomes including fetal sepsis and PTB (Boldenow et al., 2016).

In contrast to the robust proinflammatory response to hemolysin, GBS can also dampen immune responses at the maternal-fetal interface through the action of HylB, which interferes with TLR2/4-mediated immune signaling (Kolar et al., 2015). Similar to COH1Δ*covR*-infected animals, pregnant nonhuman primates choriodecidually infected with GB37, a GBS strain with high HylB activity, consistently experienced preterm labor, microbial invasion of the amniotic cavity and fetal sepsis (Coleman et al., 2021). In these experiments, neutrophils also infiltrated the chorioamniotic membranes, but were not successful in containing the infection and preventing microbial invasion of the amniotic cavity. *Ex vivo* experiments using paired maternal and neonatal cord blood indicate that HylB-mediated TLR2/4 dampening resulting in reduced production of antimicrobial reactive oxygen species (**Figure 2**, maternal/cord blood) (Coleman et al., 2021). Finally, digital spatial profiling of GBS-infected choriomaniotic membranes highlighted HylB-specific dampening of inflammatory responses (Coleman et al., 2021). This blunting of innate immune responses observed in GB37-infected animals is directly in contrast to the rapid proinflammatory response observed with the hyperhemolytic GBS (Boldenow et al., 2016). These studies highlight key virulence factor-mediated differences between two GBS strains that induce similar adverse outcomes in pregnant nonhuman primates, and emphasizes the diverse nature of GBS virulence factors.

### Placental Chorioamniotic Weakening After GBS Infection

GBS infection and inflammation of the amniotic fluid can contribute to preterm premature rupture of membranes (pPROM) through weakening of the placental chorioamniotic membranes, ultimately inducing PTB. Multiple factors contribute to membrane weakening, including matrix metalloproteinases, apoptosis, oxidative stress, and other neutrophil proteases (Lannon et al., 2014; Romero et al., 2014). An important mechanism contributing to pPROM was demonstrated using a nonhuman primate model of GBS is EMT (Weed et al., 2020). EMT is a coordinated process whereby epithelial cells change morphology, retract their cytoskeleton, lose cell-cell adhesions and become motile; overall, this process reduces tissue integrity. In a nonhuman primate model, a choriodecidual inoculation of GBS was associated with transcriptional responses in the chorioamniotic membranes characterized by downregulation of cytostructural genes and microRNA gene expression (i.e. miR-200b, miR-203-5p) consistent with EMT (Vanderhoeven et al., 2014). Immunohistochemistry of the chorioamniotic membranes also demonstrated partial loss of surface E-cadherin, increased N-cadherin expression, and vimentin staining near areas of neutrophil influx, all signs of reduced amniotic epithelial integrity (Weed et al., 2020). Thus, in addition to the role of EMT in vaginal exfoliation, EMT may also impair chorioamniotic membrane tissue integrity and predispose GBS-infected pregnant women to pPROM.

## Conclusion

In summary, GBS inflicts an annual public health burden on pregnant women and neonates, which is disproportionately concentrated in low-income countries. National and region-specific discrepancies between screening guidelines and the use of IAP confounds the accurate detection of invasive GBS disease cases, limiting the ability to control the associated adverse outcomes through intervention. Although a combination of a global universal screening approach and IAP use would result in the greatest reduction in maternal, fetal, and neonatal adverse outcomes, this is not realistic for many low-income countries that lack resources to implement these approaches; further, some higher income countries have a low prevalence of invasive GBS disease, leading them to instead adopt a more cost-effective risk-based approach to treating GBS in pregnancy. The solution to reducing invasive GBS disease in pregnant women and neonates is not simple, but the development of a GBS vaccine would be a major step forward in preventing disease.

This review highlights recent discoveries that elucidate the interplay between GBS and the host, defining the mechanisms that GBS employs to successfully disseminate to vulnerable host niches during pregnancy. In recent years, major strides have been made to improve our understanding of GBS vaginal colonization, ascending infection, and congenital infection, ultimately facilitating the strategic development of several candidate vaccines with demonstrated efficacy in animal models or human clinical trials. Although it is too early to forecast whether these vaccines will someday receive FDA-approval, their success symbolizes a major step towards reducing the global burden of GBS invasive disease.

## Author Contributions

All authors contributed to the article and approved the submitted version.

## Funding

This work was supported by funding from the National Institutes of Health grants R01AI133976, R01AI145890, R01HD098713 to LR and KW, R01AI152268 to LR., T32AI055396 to AF (PI: Fang), T32AI007509 to AB (PI: Campbell), and seed funds from Seattle Children’s Research Institute to L.R. This work was also supported by the P51OD010425, which is the core grant of the Washington National Primate Research Center. Additional support came from a U42OD011123, which funds the breeding colony at the Washington National Primate Research Center.

## Conflict of Interest

The authors declare that the research was conducted in the absence of any commercial or financial relationships that could be construed as a potential conflict of interest.

## Publisher’s Note

All claims expressed in this article are solely those of the authors and do not necessarily represent those of their affiliated organizations, or those of the publisher, the editors and the reviewers. Any product that may be evaluated in this article, or claim that may be made by its manufacturer, is not guaranteed or endorsed by the publisher.
